# HBV Envelope Protein-Bearing Vesicles Show Preferential Uptake in Hepatocyte-Derived Cells

**DOI:** 10.3390/ijms27104331

**Published:** 2026-05-13

**Authors:** Eri Takayama, Misaki Enomoto, Manami Nagae, Momoko Tomoda, Yuta Miyazumi, Yuki Iwaisako, Ryota Shirasawa, Youichi Suzuki, Takashi Nakano, Keiji Ueda, Masahiro Fujimuro

**Affiliations:** 1 Department of Cell Biology, Kyoto Pharmaceutical University, Kyoto 607-8412, Japan; t.eri000718@gmail.com (E.T.); ky21216@ms.kyoto-phu.ac.jp (M.N.); ky21208@ms.kyoto-phu.ac.jp (M.T.); ky22353@ms.kyoto-phu.ac.jp (Y.M.); yukiwaisako@gmail.com (Y.I.); 2 Analytical Technology Division, HORIBA Techno Service Co., Ltd., Kyoto 601-8305, Japan; ryota.shirasawa@horiba.com; 3 Department of Microbiology and Infection Control, Faculty of Medicine, Osaka Medical and Pharmaceutical University, Osaka 569-8686, Japan; youichi.suzuki@ompu.ac.jp (Y.S.); totoro@totoro.to (T.N.); 4 Laboratory of Biosafety Research, Faculty of Medicine, Osaka Medical and Pharmaceutical University, Osaka 569-8686, Japan; 5 Division of Virology, Graduate School of Medicine, Osaka University, Osaka 565-0871, Japan

**Keywords:** drug delivery, HBV, hepatocyte, NTCP, viral-like secretory vesicle, secretory vesicle, nanocarrier, virus-like particle

## Abstract

Controlled delivery using nanoparticle-based systems has attracted considerable attention; however, achieving cell-type specificity remains a major challenge. To address this issue, we focused on the intrinsic cell tropism of viruses. The hepatocyte tropism of hepatitis B virus (HBV) is mediated by interactions between its large envelope protein (L protein) and host factors, including the sodium taurocholate cotransporting polypeptide (NTCP). In this study, we explored viral-like secretory vesicles (VLSVs) displaying HBV spike proteins as a virus-inspired vesicle platform for hepatocyte targeting. We previously established a method for producing VLSVs from HBV L- and S-expressing HEK293T cells. In the present study, we developed an improved protocol using exosome-depleted fetal calf serum and optimized ultracentrifugation, resulting in VLSVs with comparable particle numbers and sizes but approximately tenfold higher protein content per particle. VLSVs were concentrated using a two-layer sucrose cushion, labeled with DiI, and purified by sucrose density gradient ultracentrifugation. We evaluated DiI uptake in hepatocyte-derived cells (HepG2 and Huh7), non-hepatic cells (MDA-MB231, H1299, HeLa, and Vero), and NTCP-overexpressing HepG2 cells. VLSVs showed preferential uptake in the following order: NTCP-overexpressing HepG2 > HepG2 > Huh7 > non-hepatic cells. Furthermore, removal of the N-terminal Flag tag from the L protein enhanced hepatocyte-associated uptake, suggesting the importance of preserving the native structure of the preS1 domain. While vesicle characterization and mechanistic validation remain to be further investigated, these findings provide a proof-of-concept for a virus-inspired vesicle platform exhibiting preferential uptake in hepatocyte-derived cells.

## 1. Introduction

Numerous nanoparticles with excellent physicochemical properties have been extensively developed for drug and gene delivery applications in chemotherapy and gene therapy. However, achieving both high stability and strong tissue specificity remains a major challenge in this field [1,2,3]. Drug delivery carriers such as liposomes and micelles, which can be surface-modified to target specific cells or tissues, are expected to see broader applications; nevertheless, their selectivity for target cells remains limited [4]. To address this limitation, we focused on leveraging the intrinsic cell tropism of viruses as a basis for designing targeting platforms. Hepatitis B virus (HBV) exhibits strong hepatotropism mediated by interactions between its large envelope protein (L protein) and host cell surface factors, including the sodium taurocholate cotransporting polypeptide (NTCP) [5]. This interaction enables HBV to selectively associate with hepatocytes and provides a potential framework for developing vesicle-based systems with hepatocyte-targeting properties. VLSVs are nanoscale, cell-derived secretory vesicles composed of viral spike proteins and cellular membranes. As they lack viral genomic DNA, they are expected to mimic certain viral surface properties without causing infection, and may exhibit preferential association with hepatocyte-derived cells.

HBV is a DNA virus classified under the genus *Orthohepadnavirus* of the family *Hepadnaviridae*, and is known to cause hepatitis, liver cirrhosis, and hepatocellular carcinoma in infected individuals [5,6]. The viral genome consists of partially double-stranded DNA and is enclosed within a capsid—also referred to as the core particle—composed of viral proteins, including the HBc antigen [6]. The HBV envelope is a lipid bilayer derived from endoplasmic reticulum (ER) or the Golgi membrane of human hepatocyte, which surrounds a genome-containing capsid. Embedded within this envelope are three types of surface antigens (commonly referred to as HBsAg): the large (L), middle (M), and small (S) proteins [7]. The HBV genome encodes these envelope proteins in a single open reading frame (HBsAg gene) with three independent translational start codons and a common stop codon within the HBsAg gene. This HBsAg gene can be divided into three different coding regions known as ORF preS1, ORF preS2, and ORF S [6,7,8]. S protein (226 amino acids), encoded by ORF S, consists solely of the S region. S protein is the major component of the HBV envelope. M protein carries 55 additional aa (preS2 region) at the N-terminus of S protein, and the L protein contains 108 or 119 additional aa (preS1 domain), according to the subtype, at the N-terminus of M. Thus, HBV’s three surface antigens share the same C-terminus and only differ in the length of their N-terminal regions. Interestingly, in the blood of patients during the replicative phase of HBV infection, not only infectious HBV particles (also known as Dane particles) but also a large number of subviral particles—small spherical and tubular structures primarily composed of the S protein—are detected [5,7]. The N-terminal region (preS1 domain) of L protein is responsible for hepatocyte specificity and the binding to the hepatic cell membrane protein, NTCP, which is the HBV receptor [9,10,11,12,13,14]. S protein is thought to contribute to the formation and secretion of not only HBV virions but also subviral particles [7]. The S regions within S, M, and L are anchored to the lipid bilayer membranes of viral envelopes. Although the functions of L and S proteins are characterized, the role of the M protein remains unclear [5].

HBV-derived nanoparticles have been explored for application in vaccines and delivery systems, including core particle-based carriers and L protein-based particles [15,16,17,18,19,20]. Previously, we demonstrated that the co-expression of L and S proteins in mammalian cells leads to the secretion of VLSVs and suggested their potential utility in delivery-related applications [21,22]. However, the preparation method required multiple purification steps, resulting in low yield and limited practicality.

To address these limitations, in the present study we aimed to develop a simplified preparation method for VLSVs and to evaluate their interaction with hepatocyte-derived cells. By optimizing ultracentrifugation conditions and modifying the L protein, we assessed whether VLSVs exhibit preferential uptake characteristics, particularly in NTCP-expressing cells. Importantly, the objective of this study was not to establish a fully functional drug delivery system, but rather to provide a proof-of-concept for a virus-inspired vesicle platform exhibiting hepatocyte-preferential uptake characteristics.

## 2. Results

### 2.1. Secretion of VLSV by L Protein and S Protein Overexpression

**Figure 1:**
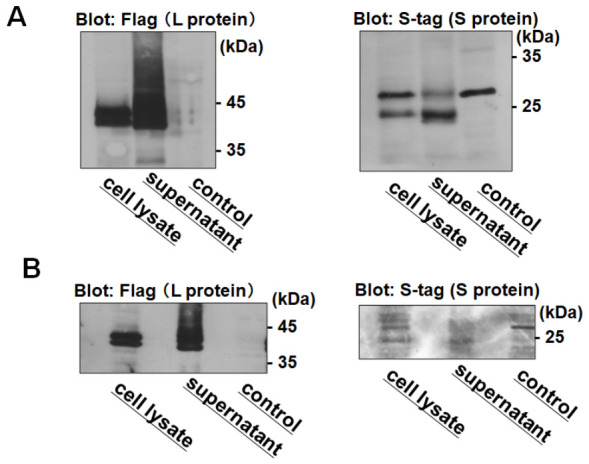
The secretion of VLSVs under different culture conditions. HEK293T cells were transfected with expression plasmids encoding Flag-tagged L protein (Flag-L) and S-tagged S protein (Stag-S). Cells were cultured either in DMEM supplemented with exosome-depleted fetal calf serum (FCS) ((**A**); new method) or in serum-free DMEM ((**B**); previous method). Culture supernatants (40 mL) were collected, and VLSVs were recovered from the supernatants by ultracentrifugation. The amounts of Flag-L and Stag-S in the VLSVs were analyzed by Western blotting (supernatant). The intracellular expression levels of Flag-L and Stag-S were also analyzed (cell lysate). Original images are shown in Supplementary Figure S2. Particle concentration, mean particle size, and protein concentration were evaluated by particle tracking analysis (PTA). Comparable particle numbers and sizes were observed between the two methods, whereas protein content per particle was increased under the new conditions.

Fetal calf serum (FCS) employed in cell culture contains a substantial amount of bovine-derived exosomes, which are co-purified with VLSVs during ultracentrifugation. Achieving the complete separation of exosomes from VLSVs remains technically challenging, and their residual presence may compromise the hepatocyte specificity of VLSVs. Therefore, in our previous method [22], we transfected HEK293T cells with HBV L protein and S protein expression plasmids, then cultured them in DMEM without FCS for 60 h, and purified VLSVs from the culture supernatant. However, serum-free culture medium causes cellular stress and reduced gene expression, so we examined the use of 30 h-cultivation with a medium containing FCS from which exosomes had been removed. Exosomes were removed from FCS by ultracentrifugation at 100,000× *g* for 20 h. HEK293T cells (6 × 10^6^ cells per dish) were seeded into four 10 cm dishes and transfected with expression plasmids encoding Flag-tagged L protein (Flag-L) and S-tagged S protein (Stag-S). To produce VLSVs, transfected cells were cultured either in 40 mL of DMEM supplemented with exosome-free FCS for 30 h or in 40 mL of serum-free DMEM for 60 h. VLSVs secreted into the culture supernatants were pelleted by ultracentrifugation, and the resulting VLSV pellets were resuspended in 1 mL of PBS. The intracellular expression levels and extracellular secretion levels of Flag-L and Stag-S were analyzed by Western blotting using anti-Flag and anti-Stag antibodies (Figure 1). Using the new approach with exosome-free FCS-containing medium (Figure 1A), both the intracellular expression and extracellular secretion of Flag-L and Stag-S were increased compared with those obtained using the previous serum-free method (Figure 1B). Furthermore, particle tracking analysis (PTA) was used to assess particle concentration and mean particle size of VLSVs prepared using the old and new protocols, and total VLSV protein concentration was determined. Although no substantial differences were observed in particle number or average particle size between the two protocols, a marked difference in protein content was detected. Specifically, the particle concentration, mean particle diameter, and protein concentration of VLSVs prepared using the new protocol were 6.1 × 10^9^ particles/mL, 140 nm, and 261 μg/mL, respectively, whereas those of VLSVs prepared using the old protocol were 8.3 × 10^9^ particles/mL, 135 nm, and 23 μg/mL, respectively (Supplementary Figure S1). Consequently, the amount of protein per particle in VLSVs prepared using the new protocol was approximately tenfold higher than that observed with the old protocol.

### 2.2. Purification of VLSV

**Figure 2:**
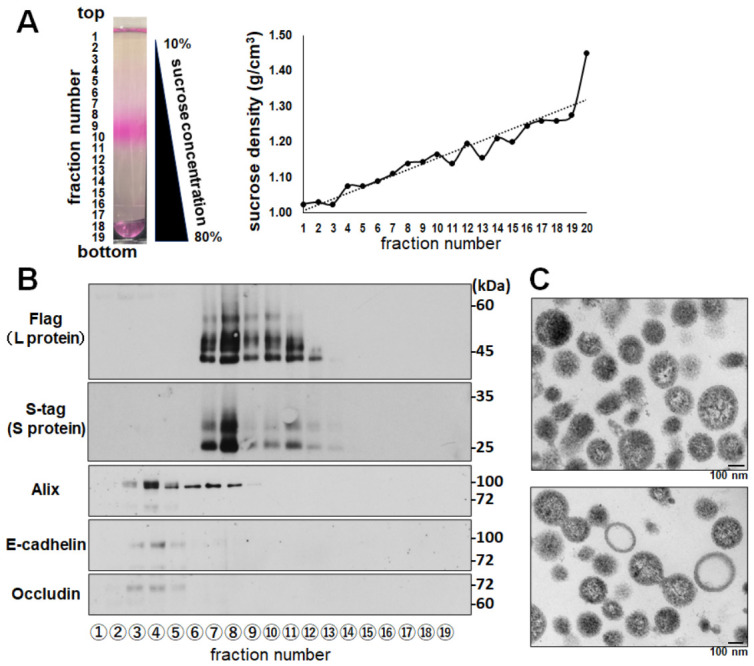
Purification and characterization of VLSVs. DiI-labeled VLSVs were purified by sucrose density gradient ultracentrifugation. (**A**) Representative image of gradient after ultracentrifugation and fraction collection. The dashed line represents the linear trendline. (**B**) Distribution of Flag-L and Stag-S proteins across fractions, as determined by Western blotting. Exosome-associated markers (Alix, E-cadherin, and occludin) were also assessed. Fractions #7–#9 contained VLSVs enriched in L and S proteins. Original images are shown in Supplementary Figure S3. (**C**) Transmission electron microscopy (TEM) images showing vesicle-like structures with diameters of approximately 100–150 nm.

Electron microscopy of VLSVs prepared by the previous protocol revealed shrunken and distorted particles [22]. This suggests that the ultracentrifugation (100,000× *g*, 2 h) in this protocol may damage VLSV structure. To overcome this issue, we developed improved purification strategies aimed at minimizing VLSV damage by shortening centrifugation time (75,000× *g* for 1 h) and introducing a two-layer sucrose cushion at the bottom of the centrifuge tube. HEK293T cells transfected with Flag-L and Stag-S expression plasmids were cultured in DMEM containing exosome-free FCS for 30 h. To precipitate VLSVs, the culture supernatant was centrifuged at 75,000× *g* for 1 h using a centrifuge tube prepared with a sucrose cushion consisting of 70% sucrose at the bottom and 20% sucrose layered on top. The VLSV precipitated between 20% and 70% sucrose cushion was stained with DiI. The DiI-labeled VLSV was loaded onto gradients of 10–80% sucrose and ultracentrifuged at 100,000× *g* for 24 h. Figure 2A shows the centrifuge tube after gradient ultracentrifugation, from which 19 fractions of 2 mL each were collected. The sucrose density of each fraction is indicated on the right. To concentrate the samples, each fraction was further ultracentrifuged at 100,000× *g* for 1 h, and the resulting pellet was resuspended in SDS-PAGE sample buffer for Western blot analysis. To identify VLSV-containing fractions, Flag-L and Stag-S proteins within VLSVs were probed using anti-tag antibodies (Figure 1B). Fractions #7–11 contained Flag-L and Stag-S proteins; notably, strong signals for both proteins were detected in fractions #7 and #8, suggesting the presence of VLSVs in these fractions. Alix, E-cadherin, and occludin are proteins commonly associated with exosomes. To assess the separation of VLSVs from exosomes, these proteins were also examined. Fractions #3 and #4 contained Alix, E-cadherin, and occludin, suggesting the partial separation of VLSVs and exosomes. However, Alix was also detected in fractions #7 and #8, which contained VLSVs, indicating that complete separation was not achieved. Moreover, since it remains unclear whether VLSVs inherently contain these markers, further analysis is required to confirm purity. VLSVs were observed by TEM analysis (Figure 2C). VLSVs presented viral-like structures (diameter about 100–150 nm). Although some empty particles or fused particles were detected, many were spherical bodies containing small inner dot structures with high electron densities. TEM presented VLSV did not present in a single morphology, this suggests that our VLSVs may be contaminated with other particles such as exosome or SVP.

**Figure 3:**
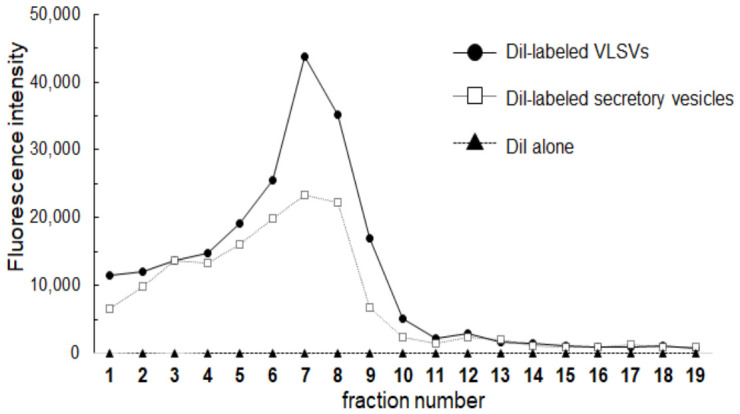
Uptake of DiI-labeled vesicles in NTCP-expressing HepG2 cells across fractions obtained by sucrose density gradient ultracentrifugation. Following sucrose gradient ultracentrifugation, 19 fractions were collected and assessed for their ability to generate DiI uptake signals in NTCP-expressing HepG2 cells using a DiI uptake assay. As a control, cellular secretory vesicles collected from the culture supernatant of empty vector-transfected cells were labeled with DiI and subjected to sucrose gradient ultracentrifugation under the same conditions as the VLSVs. As an additional control, DiI alone was also subjected to gradient ultracentrifugation under identical conditions. The DiI uptake profiles of DiI-labeled VLSVs (black circles), DiI-labeled cellular secretory vesicles (white squares), and DiI alone (black triangles) are shown.

NTCP expression is markedly downregulated in most poorly differentiated human hepatocellular carcinomas cell lines, including HepG2 and Huh7 cells [11,12,13]. Furthermore, NTCP expression is rapidly lost following the isolation of primary human hepatocytes [23]. Therefore, a stable NTCP plasmid-transfected HepG2 cell line [24] was employed as the target cell for the prepared VLSV. The 19 fractions obtained after gradient ultracentrifugation were evaluated for DiI uptake in NTCP-expressing HepG2 hepatocytes (Figure 3). As a control, cellular secretory vesicles such as exosomes collected from the culture supernatant of HEK293T cells transfected with an empty vector (pCIneo) were also purified by gradient ultracentrifugation and evaluated in the same manner as VLSVs. Specifically, the vesicles were concentrated by ultracentrifugation using a two-layer sucrose cushion, labeled with DiI, and subjected to gradient ultracentrifugation to generate 19 fractions, which were then evaluated for their DiI uptake. As an additional control, DiI alone was also subjected to gradient ultracentrifugation under identical conditions, and each fraction was evaluated in the same manner as VLSVs and cellular secretory vesicles. Fractions #7, #8, and #9 showed hepatocyte-preferential DiI uptake (Figure 3). These fractions corresponded to those presumed to contain VLSVs enriched with both Flag-L and Stag-S proteins (Figure 2B). Fractions #7, #8, and #9 were pooled (total 6 mL, sucrose concentration: 30–35%) and used as purified VLSVs for PTA and subsequent experiments. The PTA experiments showed that the particle concentration, and mean particle diameter of pooled VLSVs were 2.5 × 10^9^ particles/mL, and 136 nm, respectively (Supplementary Figure S5).

**Figure 4:**
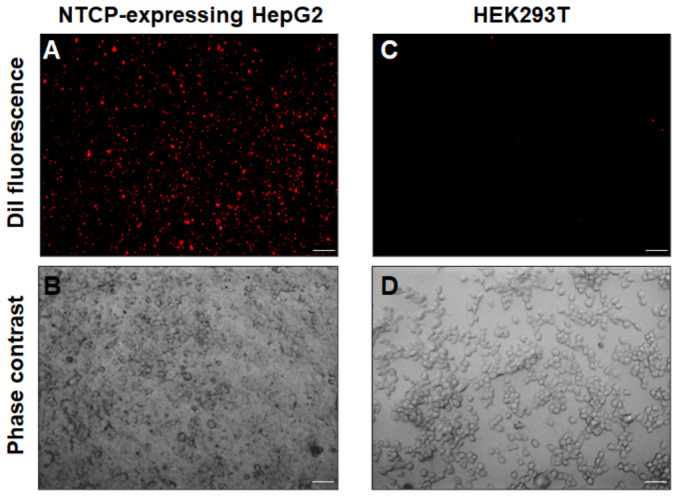
Fluorescence images of cells incorporating DiI-labeled VLSVs. NTCP-expressing HepG2 cells (**A**,**B**) or HEK293T cells (**C**,**D**) were incubated with DiI-labeled VLSVs for 2 h to allow uptake. After washing the cells twice with medium, they were incubated for 10 min and observed alive without fixation. DiI fluorescence signals (**A**,**C**) and phase-contrast images (**B**,**D**) of the cells were obtained using an inverted fluorescence microscope. Scale bars = 100 μm.

Next, the uptake of DiI into hepatocyte was evaluated by a fluorescence imaging analysis (Figure 4). The VLSV fraction was added to the culture medium of NTCP-expressing HepG2 hepatocytes or HEK293T kidney cells, and the fluorescence of DiI internalized into the cells was observed under a fluorescence microscope after 2 h. DiI uptake was clearly higher in NTCP-expressing HepG2 hepatocytes than in HEK293T cells, confirming the selective uptake characteristics of VLSVs.

### 2.3. The Cell Selectivity of Purified VLSV

**Figure 5:**
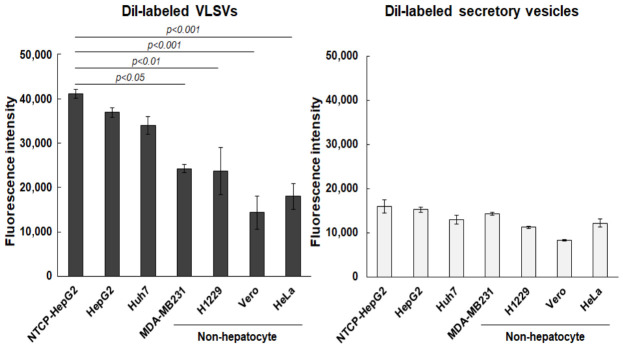
Cell-type–dependent uptake of VLSVs. The cell tropism of VLSVs was evaluated using a DiI uptake assay. Cells derived from liver (HepG2, NTCP-expressing HepG2 [NTCP-HepG2], and Huh7), mammary gland (MDA-MB231), lung (H1299), cervix (HeLa), and African green monkey kidney (Vero) were seeded at 5 × 10^4^ cells per well and treated with 5 × 10^7^ particles of DiI-labeled VLSVs for 20 min. After washing, cells were lysed, and fluorescence intensities of the lysates were measured (**left panel**). As a control (**right panel**), DiI-labeled secretory vesicles purified from the culture supernatant of empty vector-transfected cells were analyzed under the same conditions as the VLSVs. The y-axis indicates the fluorescence intensity generated when 5 × 10^7^ VLSV particles were taken up by 5 × 10^4^ cells. Autofluorescence levels of NTCP-HepG2, HepG2, Huh7, MDA-MB231, H1299, Vero, and HeLa cells were minimal compared with VLSV-treated cells, with values of 532, 293, 293, 162, 909, 185, and 296, respectively. Data are presented as mean ± SD (n = 3 independent wells). *p* < 0.05, *p* < 0.01, and *p* < 0.001 indicate statistically significant differences compared with NTCP-expressing HepG2 cells.

We next evaluated the cell tropism of purified VLSVs using a DiI uptake assay (Figure 5). Multiple cell lines derived from different tissues were tested: liver (HepG2 and Huh7), mammary gland (MDA-MB231), lung (H1299), cervix (HeLa), and African green monkey kidney (Vero). Cells were seeded in 48-well plates at 5 × 10^4^ cells per well. After removing the culture medium, 5 × 10^7^ particles of DiI-labeled VLSVs (20 µL of a mixture of fractions #7, #8, and #9) were diluted tenfold with a medium and added to each well at a final volume of 200 µL per well. The cells were incubated at 37 °C for 20 min to allow the uptake of DiI-labeled VLSVs. Cells were then washed, lysed, and the fluorescence intensities of the lysates were measured. As a control, DiI-labeled secretory vesicles purified from the culture supernatant of empty vector-transfected cells were also added into culture media and analyzed under the same conditions as the VLSVs. DiI-labeled VLSVs showed preferential uptake in the following order: NTCP-overexpressing HepG2 > HepG2 > Huh7 > non-hepatic cells (MDA-MB231 > H1299 > HeLa > Vero). Compared with VLSVs, secretory vesicles including exosomes showed no significant uptake specificity among the cells tested. NTCP is essential for HBV attachment and entry into host hepatocytes. It is well known that NTCP expression is reduced in poorly differentiated hepatocellular carcinomas cells such as HepG2 and Huh7 [11,12,13]. These previous findings support our observation that VLSVs bearing the L protein preferentially uptake in NTCP-overexpressing HepG2 cells compared to parental HepG2 cells. However, the uptake efficiency of VLSVs in NTCP-overexpressing HepG2 cells and NTCP-low hepatocyte-derived cells (HepG2 and Huh7) was not markedly higher than that observed for non-hepatic cell lines.

### 2.4. Effect of N-Terminal Tag Modification of L Protein on VLSV Selectivity and Uptake

**Figure 6:**
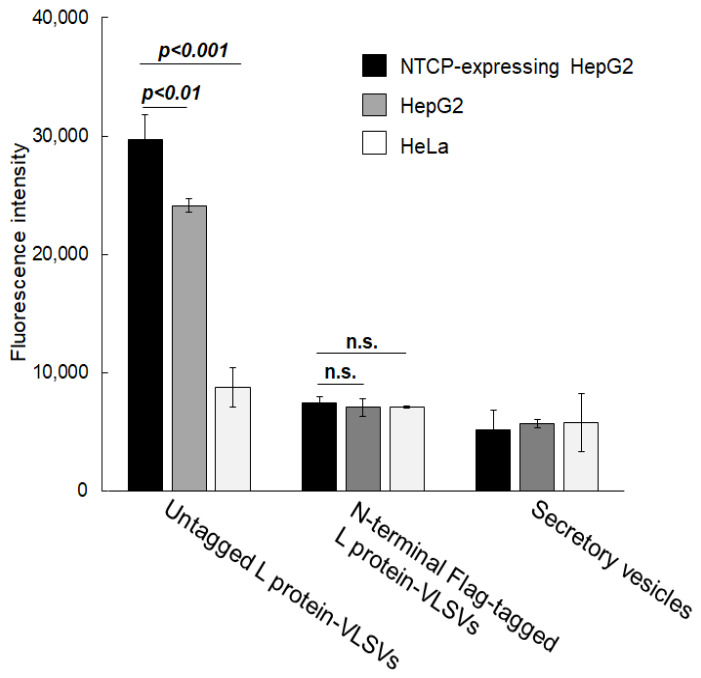
Effect of N-terminal modification of the L protein on VLSV uptake. To assess the impact of N-terminal Flag-tagging of the L protein on VLSV uptake, we compared VLSVs containing Flag-tagged L protein with those containing untagged L protein. HEK293T cells were co-transfected with either N-terminal Flag-tagged or untagged L protein together with S-tagged S protein, and DiI-labeled VLSVs were purified from the culture supernatant using sucrose gradient centrifugation. As a control, DiI-labeled secretory vesicles were purified from the supernatant of empty vector-transfected cells under the same conditions as VLSVs. Flag-tagged L protein-VLSVs (5 × 10^7^ particles) or exosomes were then added to 5 × 10^4^ cells of NTCP-expressing HepG2 cells, HepG2 cells, and HeLa cells, and DiI uptake efficiency was quantified. The y-axis indicates the fluorescence intensity generated when 5 × 10^7^ VLSV particles were taken up by 5 × 10^4^ cells. Data are presented as mean ± SD (n = 3 independent wells). *p* < 0.01 and *p* < 0.001 indicate statistically significant differences compared with NTCP-expressing HepG2 cells. n.s., not significant.

The N-terminal region (preS1 domain) of the L protein is important for HBV attachment and entry into NTCP-expressing hepatocytes [11,12]. Furthermore, HBV infection was reported to be inhibited by antibodies targeting the preS1 domain [9] or by synthetic peptides containing the preS1 domain [10]. We thought that the addition of a Flag tag to the N-terminus of the L protein might impair the selective uptake of VLSVs into NTCP-expressing hepatocytes. Therefore, we compared VLSVs containing Flag-tagged L protein with those containing untagged L protein (Figure 6). HEK293T cells were co-transfected with either Flag-tagged or untagged L protein together with S-tagged S protein, cultured for 30 h in DMEM containing exosome-free FCS, and VLSVs were purified by short ultracentrifugation using a two-layer sucrose cushion, followed by DiI labeling and sucrose gradient centrifugation. Under the same conditions as in Figure 5, the two types of DiI-labeled VLSVs were added to NTCP-expressing HepG2 cells, parental HepG2 cells, and HeLa cells, and DiI uptake efficiency was measured. VLSVs containing untagged L protein exhibited significantly higher uptake to NTCP-expressing HepG2 cells and parental HepG2 cells compared to VLSVs containing Flag-L protein. These findings suggest that the presence of an N-terminal Flag tag may interfere with the interaction between L protein and NTCP, thereby reducing hepatocyte selectivity. Thus, N-terminal modifications of the L protein may negatively affect hepatocyte-associated uptake, suggesting the importance of preserving the native structure of the preS1 domain for optimal hepatocyte targeting.

## 3. Discussion

In this study, we established an improved method for generating viral-like secretory vesicles (VLSVs) bearing HBV envelope proteins and evaluated their interaction with multiple cell types. The results indicated that VLSVs prepared using exosome-depleted fetal calf serum and a two-layer sucrose cushion showed improved yield and structural integrity compared to our previous method [22]. Electron microscopy confirmed the preservation of vesicular morphology, and Western blot analysis verified the presence of both L and S proteins in VLSVs. Functional assays revealed that DiI-labeled VLSVs exhibited higher uptake in NTCP-overexpressing HepG2 cells than in parental HepG2 and Huh7 cells, indicating preferential interaction with hepatocyte-derived cells. However, the difference in uptake compared to non-hepatic cells was limited, suggesting that additional factors may influence overall selectivity.

In the initial step of HBV attachment, the virus associates with heparan sulfate proteoglycans (HSPGs) on the hepatocyte surface through low-affinity interactions [25,26]. Subsequently, the myristoylated preS1 domain of the HBV L protein binds with high affinity to NTCP, facilitating viral entry [5,11,27]. In addition, recent studies have shown that epidermal growth factor receptor (EGFR) and E-cadherin contribute to HBV internalization by forming a complex with NTCP [28,29]. These findings suggest that multiple host factors beyond NTCP may influence HBV entry. Therefore, the expression levels and interactions of these factors in hepatocyte-derived cell lines may contribute to the observed uptake patterns of VLSVs.

Mechanistically, our findings also provide insight into structural requirements for efficient cell association. We observed that N-terminal modification of the L protein with a Flag tag significantly reduced uptake in hepatocyte-derived cells. Given that N-terminal myristoylation of the preS1 domain is essential for interaction with NTCP [13,30,31], the presence of a Flag tag at this region likely interferes with receptor binding through steric hindrance or the disruption of lipid modification. Structural studies have shown that the N-terminal residues of preS1 insert into the binding pocket of NTCP and form stabilizing interactions [27]. Thus, the preservation of the native N-terminal structure appears to be critical for maintaining hepatocyte-targeting characteristics. These observations have important implications for the design of virus-inspired vesicle systems.

Despite these findings, several important limitations should be considered. First, the identity and purity of the vesicle preparations cannot be definitively established. The detection of exosome-associated markers in VLSV fractions suggests the presence of heterogeneous extracellular vesicle populations, and complete separation from exosomes was not achieved. It also remains unclear whether some of these marker proteins are inherent components of VLSVs. Such heterogeneity may influence uptake measurements and contribute to the observed variability in cell selectivity.

Second, although preferential uptake in NTCP-expressing cells was observed, the direct mechanistic involvement of NTCP remains to be experimentally validated. No receptor-blocking or competition assays were performed, and therefore the contribution of NTCP should be regarded as suggestive rather than conclusive. In addition, the overall differences in uptake between hepatocyte-derived and non-hepatic cells were modest, indicating that non-specific interactions and additional host factors may play a role.

Third, the present study relies on DiI membrane dye uptake to assess vesicle–cell interaction. While this approach is useful for evaluating relative association, it does not demonstrate the functional delivery of biologically active cargo. Therefore, the current findings should be interpreted as evidence of preferential uptake rather than confirmed intracellular delivery.

Taken together, this study provides a proof-of-concept demonstration of a virus-inspired vesicle platform exhibiting preferential uptake in hepatocyte-derived cells, while highlighting key factors that influence vesicle–cell interactions and design considerations for future development. Further studies will be required to improve vesicle purification, clarify the molecular mechanisms underlying targeting, and establish functional delivery capabilities.

## 4. Material and Methods

### 4.1. Reagents and Cells

1,1′-dioctadecyl-3,3,3′,3′-tetramethylindocarbocyanine perchlorate (DiI) was purchased from FUJIFILM Wako (Osaka, Japan) and was dissolved in ethanol. Human hepatocellular carcinoma cells (Huh-7, RCB1366), human cervical carcinoma cells (HeLa, RCB0007) and African green monkey kidney cells (Vero, RCB0001) were provided by the RIKEN BioResource Research Center (https://web.brc.riken.jp/ja/, accessed on 11 December 2025). Human hepatocellular carcinoma cells (HepG2), human embryonic kidney cells (HEK293T), human mammary gland adenocarcinoma cells (MDA-MB-231), and human lung carcinoma cells (H1299) were kindly provided by Dr. H. Ariga (School of Pharmaceutical Sciences, Hokkaido University, Sapporo, Japan). These cell lines were cultured in DMEM (high glucose) supplemented with 10% fetal calf serum (FCS). HepG2 cells stably expressing NTCP were generously provided by Dr. K. Watashi [24] and maintained in DMEM (high glucose) containing 20% FCS and 500 µg/mL G418 (Nacalai Tesque, Kyoto, Japan). All cells were cultured in a humidified CO_2_ incubator at 37 °C with 95% air and 5% CO_2_. Huh-7, HeLa, Vero, HepG2, HEK293T, MDA-MB-231, and H1299 cells were maintained in 10% FCS-containing DMEM medium without antibiotics. Only NTCP-stable expressing HepG2 cells were maintained in a medium supplemented with G418 for the selection of transformed cells, but 48 h before use in experiments, the medium was changed to 10% FCS-containing DMEM medium without antibiotics to bring the culture conditions to the same as the other cells.

### 4.2. VLSV Secretion from HEK293T Cells Expressing L and S Proteins

HEK293T cells (3 × 10^6^ cells per dish) were seeded into twenty-four 10 cm dishes and cultured at 37 °C with 95% air and 5% CO_2_ for 12 h to form a cell monolayer. Cells were transfected using the Chen-Okayama calcium phosphate method [32]. Each dish contained 3 × 10^6^ cells 8 µg of Flag-tagged L protein (Flag-L) and 8 µg of S-tagged HBV S protein (Stag-S) expression plasmids [22] were used for transfection and cultured in DMEM containing 10% FCS for 15 h. Culture media were changed to new DMEM with exosome-free FCS, and cells were further cultured for 30 h for VLSVs secretion. The culture supernatant containing VLSVs was collected and centrifuged at 1400 rpm for 5 min, then the supernatant was centrifuged at 9000 rpm for 30 min to remove debris. Exosome-depleted FCS was prepared as follows: FCS was ultracentrifuged at 100,000× *g* for 20 h to precipitate exosomes.; The supernatant of FCS was passed through 0.2 µm filter for sterilizing.

### 4.3. Purification of VLSV and Fluorescence Labeling of VLSV

The culture supernatant obtained from HEK293T cells (2.4 × 10^7^ cells) transfected with Flag-L and Stag-S expression plasmids was ultracentrifuged at 75,000× *g* for 1 h using a Optima XE-100 with an SW32Ti swing rotor (Beckman Coulter, Inc., Brea, CA, USA). For this ultracentrifugation, a 38.5 mL centrifuge tube was used, in which 1 mL of 70% sucrose was placed at the bottom, and 4 mL of 20% sucrose was layered on top of the 70% sucrose. After centrifugation, 1 mL of VLSVs was collected from the interface between the 70% and 20% sucrose layers. The recovered VLSV solution was incubated with the lipophilic fluorescent dye DiI (final concentration: 20 mM) under stirring at 4 °C for 1 h in light-shielded conditions. To remove deposit DiI, the reaction mixture was centrifuged at 9000 rpm for 30 min, and the supernatant was collected. To eliminate unbound DiI, the supernatant was ultracentrifuged at 75,000× *g* for 30 min using an SW55Ti swing rotor (Beckman Coulter, Inc.) and a 5 mL centrifuge tube prepared with the same sucrose layering. DiI-labeled VLSVs (0.5 mL) were collected from the interface between the 70% and 20% sucrose layers. The DiI-labeled VLSVs (0.5 mL) were then loaded onto a 10–80% sucrose gradient in PBS and ultracentrifuged at 100,000× *g* for 24 h. The sucrose gradient was prepared through the stepwise layering of 4.5 mL each of 80% (bottom), 70%, 60%, 50%, 40%, 30%, 20%, and 10% (top) sucrose in a 38.5 mL centrifuge tube. After centrifugation, 2 mL fractions were collected sequentially from the top of the tube, resulting in a total of 19 samples.

### 4.4. Western Blotting and Antibodies

Each of the 19 fractions (2 mL) obtained from sucrose gradient ultracentrifugation was ultracentrifuged at 100,000× *g* for 1 h using SW55Ti swing rotor. The precipitation samples were mixed with 4× SDS-PAGE sample buffer and were subjected to SDS-PAGE, followed by Western blotting. Western blotting was performed as previously described [33]. The primary antibodies anti-Flag mAb (M185) (MBL, Nagoya, Japan), anti-Stag-HRP pAb (ab18589) (Abcam, Cambridge, CA, USA), anti-GFP pAb (sc-8334), and anti-beta-actin mAb (sc-69879) (Santa Cruz Biotechnology, Santa Cruz, CA, USA) were used. As a secondary antibody, biotin-conjugated anti-mouse (or rabbit) IgG (Vector Laboratories, Inc., Newark, CA, USA) was used. The membrane treated with the secondary antibody was incubated with PBS containing the horse radish peroxidase (HRP)-biotin and avidin complex (VECTASTAIN Elite ABC Kit, PK-6102; Vector Laboratories, PA, USA). The ECL Western Blotting Detection Reagents (GE Healthcare Life Sciences, Chicago, IL, USA) were used for the visualization of the detected signal.

### 4.5. DiI Uptake Assay

Cells were seeded in 48-well plates at 5 × 10^4^ cells per well (200 µL) and cultured for 24 h. After removing the culture medium, each fraction obtained by sucrose gradient ultracentrifugation was diluted tenfold with a medium and added to each well at 200 µL/well. The cells were incubated at 37 °C for 20 min to allow the uptake of DiI-labeled VLSV. Subsequently, the cells were washed twice with PBS. Then, 100 µL of 0.2% NP-40 was added to each well to lyse the cells, and the fluorescence intensity (Ex: 545 nm, Em: 575nm) derived from DiI in the cell lysates was measured using a microplate spectrophotometer (Tecan M200; Tecan, Kawasaki, Kanagawa, Japan).

### 4.6. Fluorescence Imaging Analysis

NTCP-expressing HepG2 cells or HEK293T cells were cultured for 2 h with the DiI-labeled VLSV-containing fraction separated by a gradient ultracentrifugation. After uptake of VLSVs, the cells were washed with the culture media. Subsequently, the DiI-fluorescence signals and the phase-contrast images of living cells were obtained under an inverted microscope (IX-71; Olympus Corporation, Tokyo, Japan) with the 10X objective using analysis software DP2-BSW version 2.2 (Olympus Corporation).

### 4.7. Transmission Electron Microscopy (TEM)

The embedding of samples (n = 3), preparation of ultrathin sections and TEM analysis were performed as previously described [34]. Briefly samples were centrifuged at 100,000× *g* for 30 min, and precipitates were fixed with 2% glutaraldehyde for 2 h and 1% osmium tetroxide for 2 h.

### 4.8. Particle Tracking Analysis (PTA)

The particle size and particle concentration of VLSVs were analyzed using a ViewSizer 3000 system (HORIBA, Kyoto, Japan), which is based on particle tracking analysis (PTA). VLSV samples were diluted with PBS and loaded into a cuvette for measurement. PTA was performed under two different sets of parameters, as described below. For the measurements shown in Figure 1 and Supplementary Figure S1A–C, data were acquired using the following parameters: frame rate, 30 frames/sec; exposure time, 24 msec; gain, 24; blue laser power, 70 mW; green laser power, 12 mW; red laser power, 8 mW; and temperature, 22 °C. A total of 25 videos were recorded, with 3 s of stirring between each acquisition to ensure independent particle populations. Particle size and concentration were calculated within a size range of 50–500 nm. For the measurements shown in Figure 3 and Figure 5, and Supplementary Figure S4, data were acquired using the following parameters: frame rate, 30 frames/sec; exposure time, 32 msec; gain, 32; blue laser power, 150 mW; green laser power, 12 mW; red laser power, 8 mW; and temperature, 22 °C. As above, 25 videos were recorded with 3 s of stirring between acquisitions, and particle size and concentration were calculated within a size range of 50–500 nm.

### 4.9. Statistics

Statistical analyses were performed using Microsoft Excel^®^ and GraphPad Prism 7 (GraphPad Software, La Jolla, CA, USA). Differences between each experimental group and the control group were analyzed by one-way analysis of variance (ANOVA), followed by Dunnett’s post hoc test. *p* values are indicated in each figure. Data are presented as the mean ± standard deviation (SD), calculated from at least three independent experiments.

## 5. Conclusions

In this study, we developed an improved method for generating HBV envelope protein-bearing viral-like secretory vesicles (VLSVs) and evaluated their interaction with various cell types. VLSVs prepared under optimized conditions showed preferential uptake in hepatocyte-derived cells, with enhanced uptake observed in NTCP-expressing HepG2 cells. Although vesicle identity, targeting mechanisms, and functional delivery capacity remain to be fully elucidated, these findings provide a proof-of-concept for a virus-inspired vesicle platform exhibiting hepatocyte-preferential uptake properties. Further studies will be required to establish vesicle purity, define the molecular basis of cell targeting, and demonstrate the functional delivery of therapeutic cargo.

## Data Availability

The original contributions presented in this study are included in the article/Supplementary Material. Further inquiries can be directed to the corresponding author.

## Supplementary Materials

The following supporting information can be downloaded at: https://www.mdpi.com/article/10.3390/ijms27104331/s1.

## Abbreviations

DDS, drug delivery systems; FCS, fetal calf serum; HBV, Hepatitis B virus; L protein, large envelope protein; NTCP, sodium taurocholate transporting polypeptide; PTA, particle tracking analysis; TEM, transmission electron microscopy; VLSVs, viral-like secretory vesicles.
