# Supplementary material for: HBV Envelope Protein-Bearing Vesicles Show Preferential Uptake in Hepatocyte-Derived Cells
Source: Int J Mol Sci. 2026 May 13;27(10):4331. doi: 10.3390/ijms27104331 (PMC13207099; doi:10.3390/ijms27104331)
Supplement: Supplementary file 1 [file ijms-27-04331-s001.zip › ijms-4079703-supplementary.pdf]

Supplementary Figure S1

Fig 1 raw data of Particle tracking analysis (PTA) using ViewSizer 3000

A. Particle concentration and particle size of VLSV prepared by new protocol

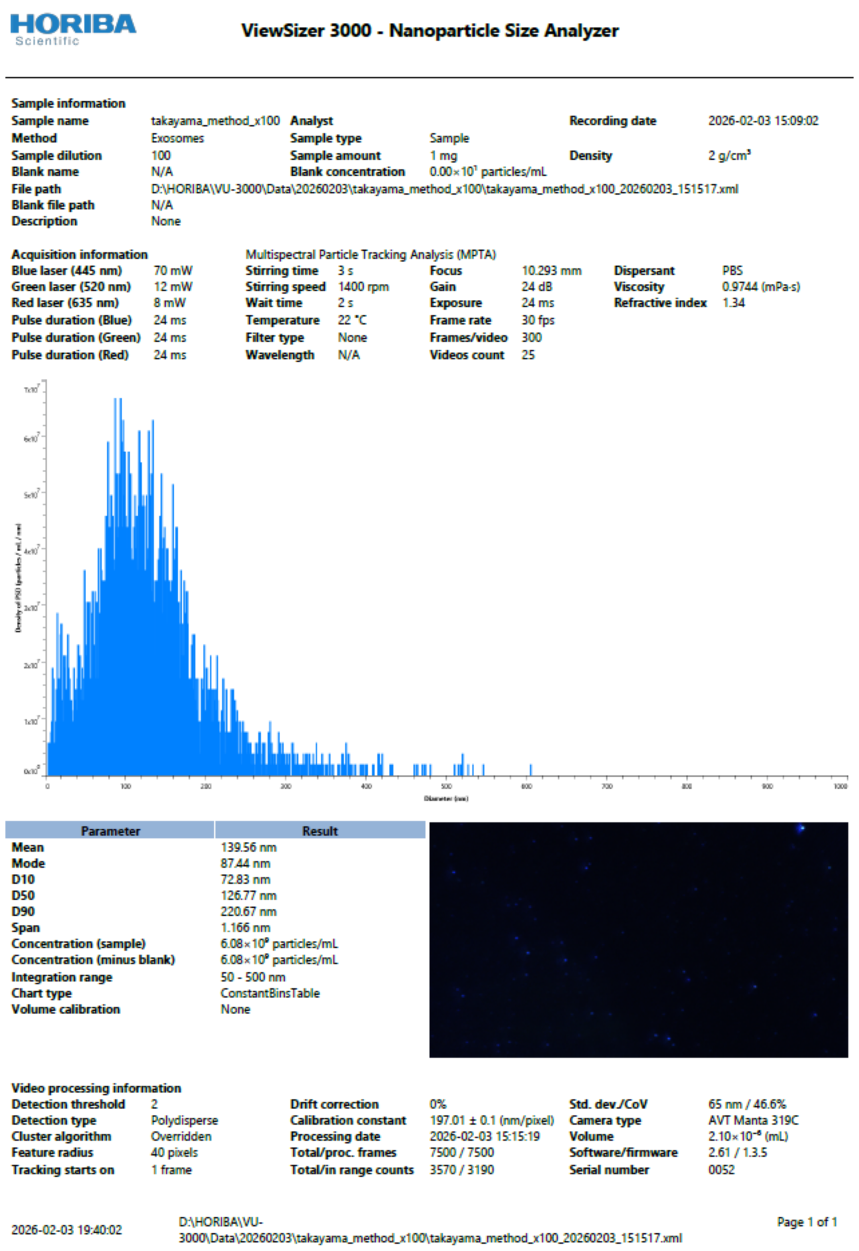

ViewSizer 3000 - Nanoparticle Size Analyzer

Sample information

**Sample name**	takayama_method_x100	**Analyst**		**Recording date**	2026-02-03 15:09:02
**Method**	Exosomes	**Sample type**	Sample		
**Sample dilution**	100	**Sample amount**	1 mg	**Density**	2 g/cm³
**Blank name**	N/A	**Blank concentration**	0.00×10^1 particles/mL		

File path D:\HORIBA\VU-3000\Data\20260203\takayama_method_x100\takayama_method_x100_20260203_151517.xml
Blank file path N/A
Description None

Acquisition information Multispectral Particle Tracking Analysis (MPTA)

**Blue laser (445 nm)**	70 mW	**Stirring time**	3 s	**Focus**	10.293 mm	**Dispersant**	PBS
**Green laser (520 nm)**	12 mW	**Stirring speed**	1400 rpm	**Gain**	24 dB	**Viscosity**	0.9744 (mPa·s)
**Red laser (635 nm)**	8 mW	**Wait time**	2 s	**Exposure**	24 ms	**Refractive index**	1.34
**Pulse duration (Blue)**	24 ms	**Temperature**	22 °C	**Frame rate**	30 fps		
**Pulse duration (Green)**	24 ms	**Filter type**	None	**Frames/video**	300		
**Pulse duration (Red)**	24 ms	**Wavelength**	N/A	**Videos count**	25		

Parameter	Result
Mean	139.56 nm
Mode	87.44 nm
D10	72.83 nm
D50	126.77 nm
D90	220.67 nm
Span	1.166 nm
Concentration (sample)	6.08×10^9 particles/mL
Concentration (minus blank)	6.08×10^9 particles/mL
Integration range	50 - 500 nm
Chart type	ConstantBinsTable
Volume calibration	None

Video processing information

**Detection threshold**	2	**Drift correction**	0%	**Std. dev./CoV**	65 nm / 46.6%
**Detection type**	Polydisperse	**Calibration constant**	197.01 ± 0.1 (nm/pixel)	**Camera type**	AVT Manta 319C
**Cluster algorithm**	Overridden	**Processing date**	2026-02-03 15:15:19	**Volume**	2.10×10^{-6} (mL)
**Feature radius**	40 pixels	**Total/proc. frames**	7500 / 7500	**Software/firmware**	2.61 / 1.3.5
**Tracking starts on**	1 frame	**Total/in range counts**	3570 / 3190	**Serial number**	0052

2026-02-03 19:40:02 D:\HORIBA\VU-3000\Data\20260203\takayama_method_x100\takayama_method_x100_20260203_151517.xml Page 1 of 1

B. Particle concentration and particle size of VLSV prepared by old protocol

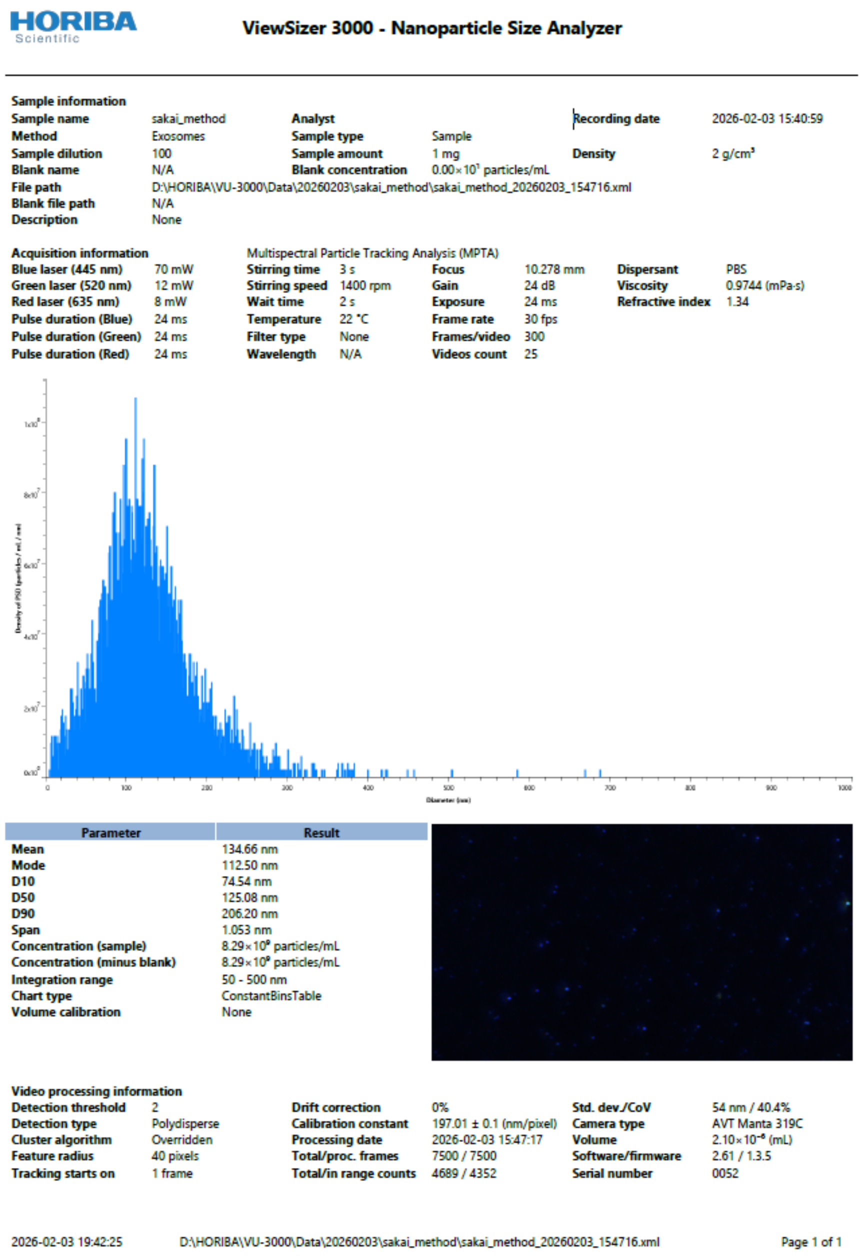

ViewSizer 3000 - Nanoparticle Size Analyzer

Sample information

**Sample name**	sakai_method	**Analyst**		**Recording date**	2026-02-03 15:40:59
**Method**	Exosomes	**Sample type**	Sample		
**Sample dilution**	100	**Sample amount**	1 mg	**Density**	2 g/cm³
**Blank name**	N/A	**Blank concentration**	0.00×10^1 particles/mL		

File path D:\HORIBA\VU-3000\Data\20260203\sakai_method\sakai_method_20260203_154716.xml
Blank file path N/A
Description None

Acquisition information Multispectral Particle Tracking Analysis (MPTA)

**Blue laser (445 nm)**	70 mW	**Stirring time**	3 s	**Focus**	10.278 mm	**Dispersant**	PBS
**Green laser (520 nm)**	12 mW	**Stirring speed**	1400 rpm	**Gain**	24 dB	**Viscosity**	0.9744 (mPa·s)
**Red laser (635 nm)**	8 mW	**Wait time**	2 s	**Exposure**	24 ms	**Refractive index**	1.34
**Pulse duration (Blue)**	24 ms	**Temperature**	22 °C	**Frame rate**	30 fps		
**Pulse duration (Green)**	24 ms	**Filter type**	None	**Frames/video**	300		
**Pulse duration (Red)**	24 ms	**Wavelength**	N/A	**Videos count**	25		

Parameter	Result
Mean	134.66 nm
Mode	112.50 nm
D10	74.54 nm
D50	125.08 nm
D90	206.20 nm
Span	1.053 nm
Concentration (sample)	8.29×10^9 particles/mL
Concentration (minus blank)	8.29×10^9 particles/mL
Integration range	50 - 500 nm
Chart type	ConstantBinsTable
Volume calibration	None

Video processing information

**Detection threshold**	2	**Drift correction**	0%	**Std. dev./CoV**	54 nm / 40.4%
**Detection type**	Polydisperse	**Calibration constant**	197.01 ± 0.1 (nm/pixel)	**Camera type**	AVT Manta 319C
**Cluster algorithm**	Overridden	**Processing date**	2026-02-03 15:47:17	**Volume**	2.10×10^{-6} (mL)
**Feature radius**	40 pixels	**Total/proc. frames**	7500 / 7500	**Software/firmware**	2.61 / 1.3.5
**Tracking starts on**	1 frame	**Total/in range counts**	4689 / 4352	**Serial number**	0052

2026-02-03 19:42:25 D:\HORIBA\VU-3000\Data\20260203\sakai_method\sakai_method_20260203_154716.xml Page 1 of 1

C. The overlay of data A (new protocol) and data B (old protocol)

ViewSizer 3000 - Nanoparticle Size Analyzer

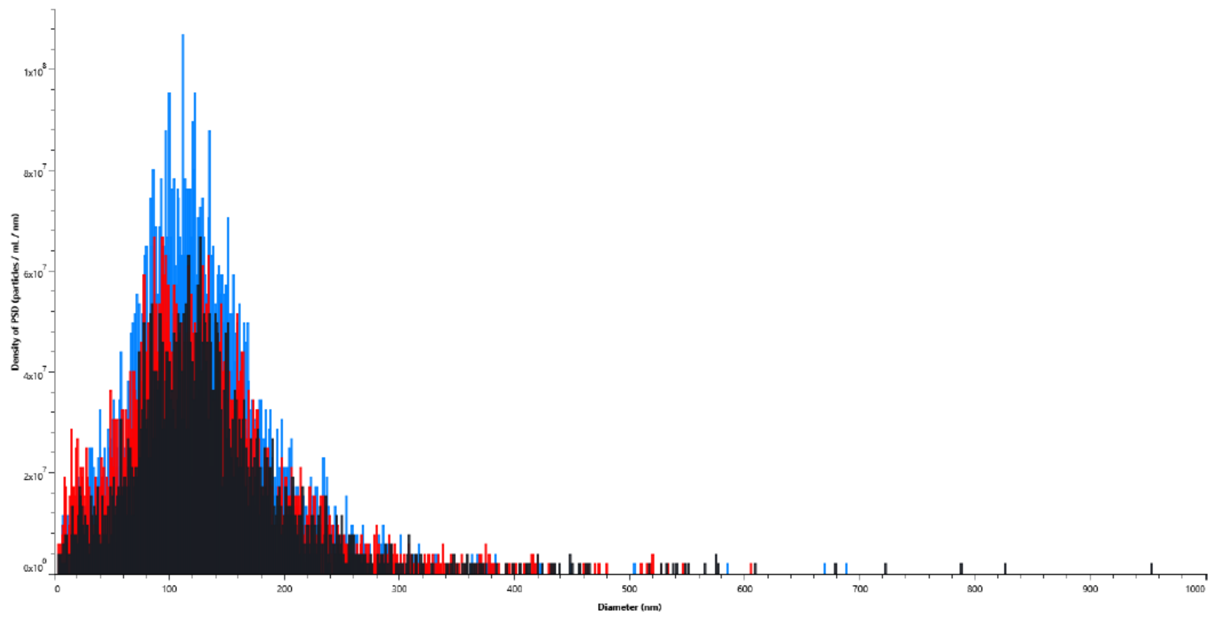

Sample	Color	Mode	Total counts	Mode (nm)	D50 (nm)	D10 (nm)	D90 (nm)	Span (nm)	Concentration (particles/mL)
sakai_method		MPTA	4352	112.50	125.08	74.54	206.20	1.053	8.29×10^9
takayama_method_x100		MPTA	3190	87.44	126.77	72.83	220.67	1.166	6.08×10^9
supernatant		MPTA	2764	127.56	128.14	77.26	212.40	1.055	5.26×10^9
		Average	3435.33	109.17	126.66	74.88	213.09	1.09	6.54×10^9
		CoV (%)	23.93	18.56	1.21	2.98	3.41	5.93	23.93

Supplementary Figure S2

Original Data of Fig. 1A and 1B

A

Blot: Flag（L protein）

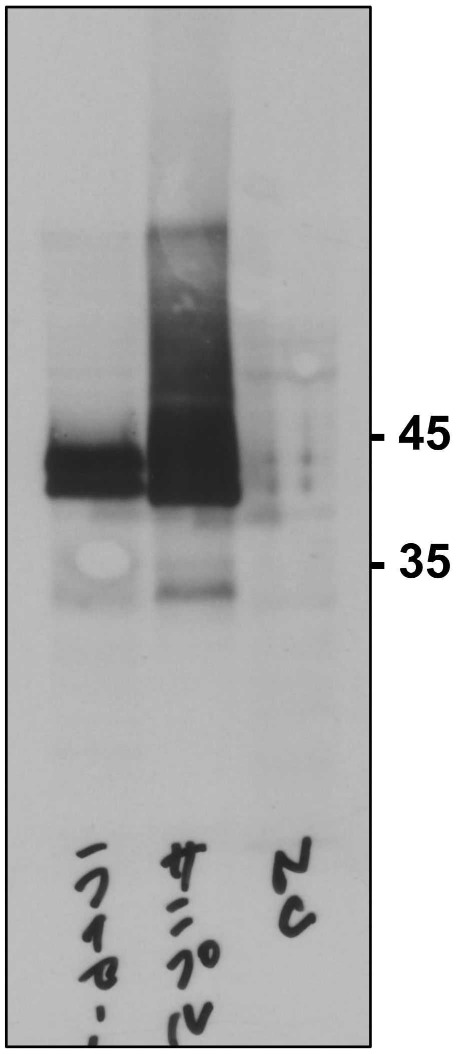

Blot: S-tag (S protein)

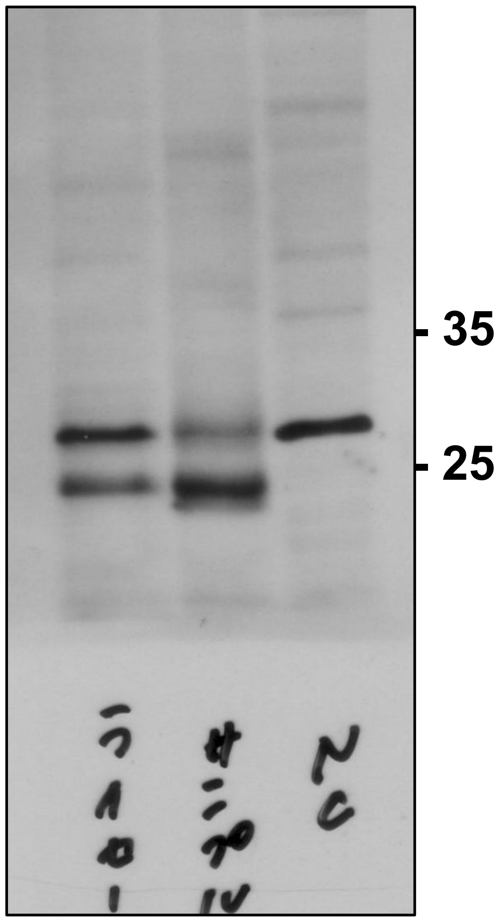

B

Blot: Flag（L protein）

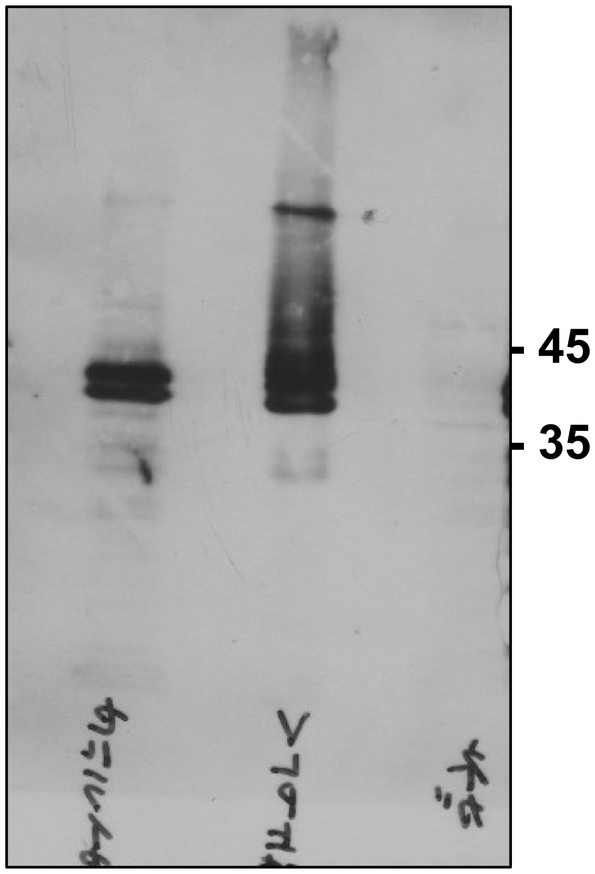

Blot: S-tag (S protein)

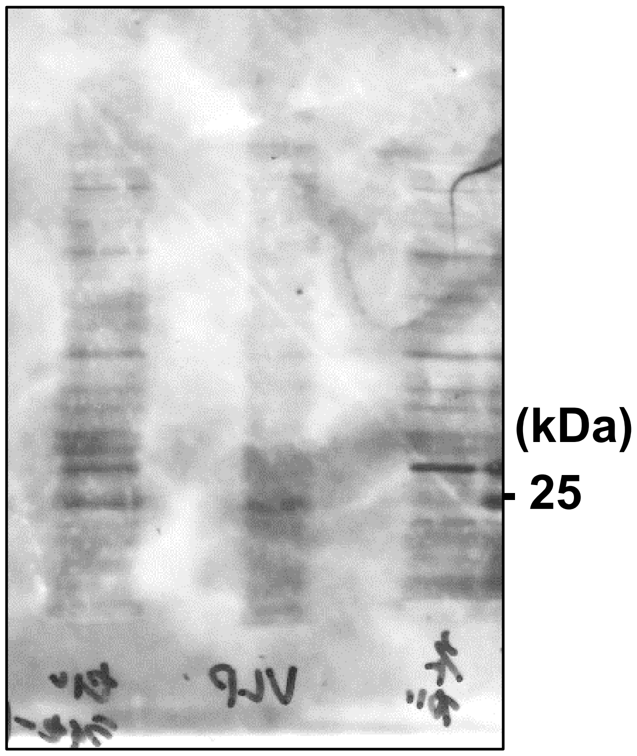

Supplementary Figure S3

Original Data of Fig. 2B

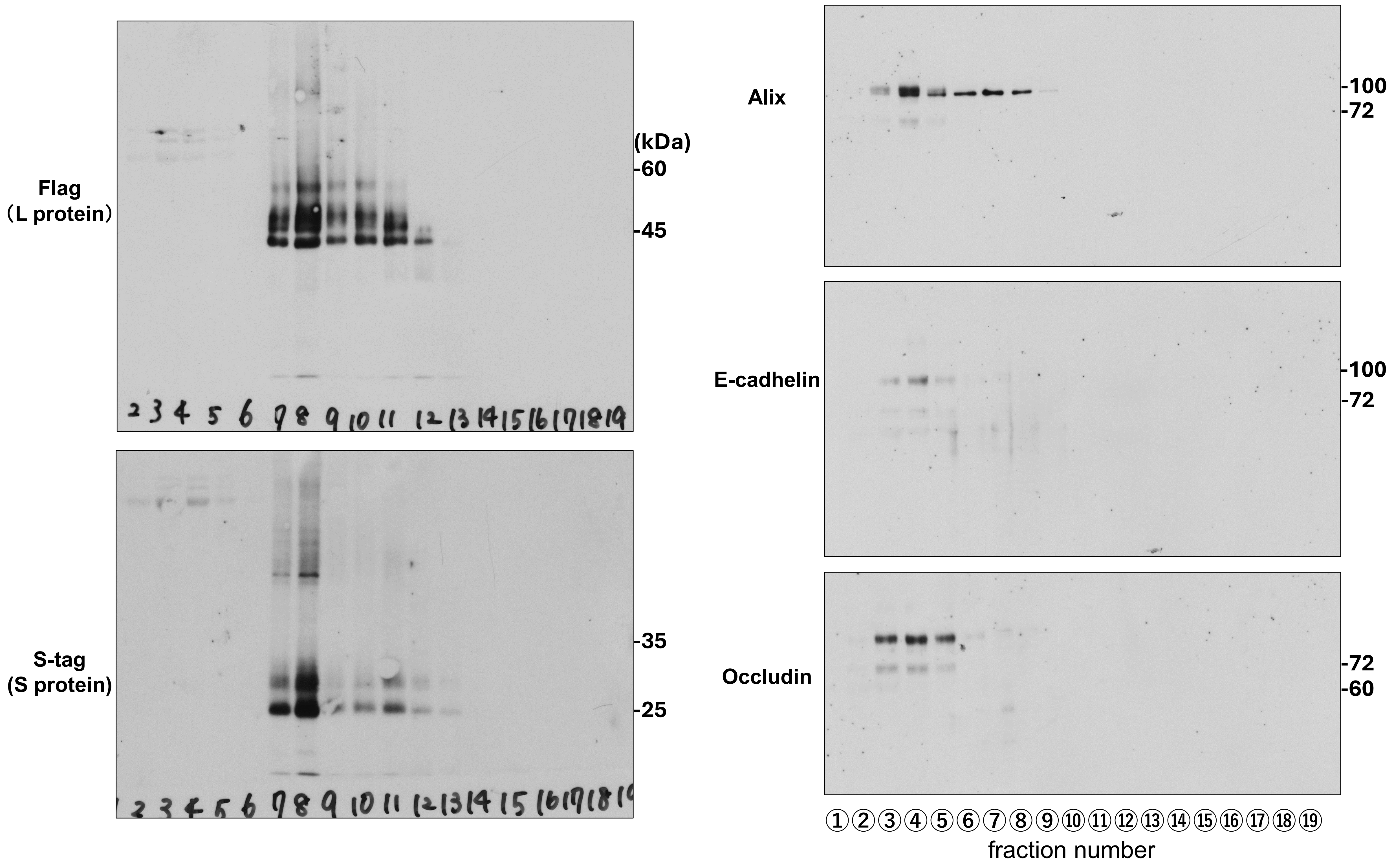

Supplementary Figure S4

Original Data of Fig. 2C

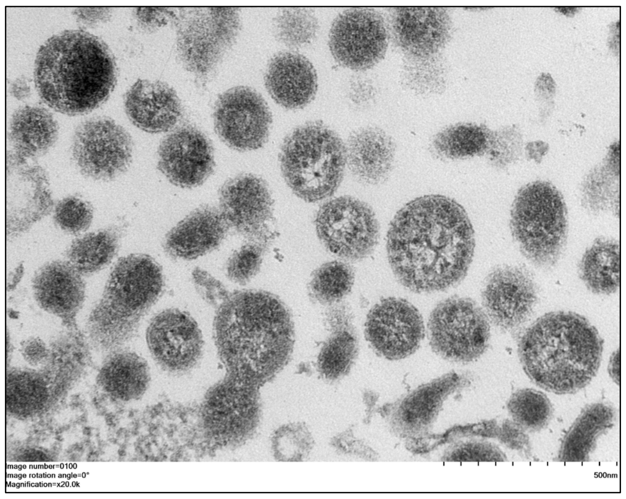

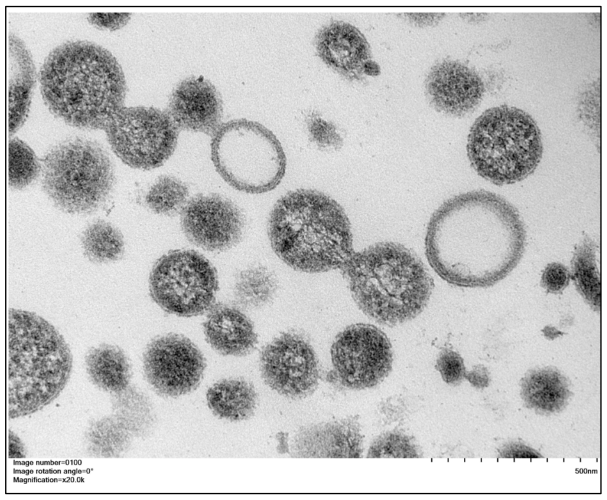

Supplementary Figure S5

Raw data of Particle tracking analysis (PTA) using ViewSizer 3000 in Fig 3 and Fig 5

Particle concentration and particle size of VLSV

HORIBA Scientific

ViewSizer 3000 - Nanoparticle Size Analyzer

Sample information

**Sample name**	#7-9	**Analyst**		**Recording date**	2026-03-19 14:22:05
**Method**	20260319_MPTA	**Sample type**	Sample		
**Sample dilution**	50	**Sample amount**	1 mg	**Density**	2 g/cm^3
**Blank name**	N/A	**Blank concentration**	0.00×10^{1} particles/mL		
**File path**	D:\HORIBA\VU-3000\Data\20260319_demo\#7-9\#7-9_20260319_142821.xml				
**Blank file path**	N/A				
**Description**	None				

Acquisition information — Multispectral Particle Tracking Analysis (MPTA)

**Blue laser (445 nm)**	150 mW	**Stirring time**	3 s	**Focus**	10.309 mm	**Dispersant**	PBS
**Green laser (520 nm)**	12 mW	**Stirring speed**	1400 rpm	**Gain**	32 dB	**Viscosity**	0.9744 (mPa·s)
**Red laser (635 nm)**	8 mW	**Wait time**	2 s	**Exposure**	32 ms	**Refractive index**	1.34
**Pulse duration (Blue)**	32 ms	**Temperature**	22 °C	**Frame rate**	30 fps		
**Pulse duration (Green)**	32 ms	**Filter type**	None	**Frames/video**	300		
**Pulse duration (Red)**	32 ms	**Wavelength**	N/A	**Videos count**	25		

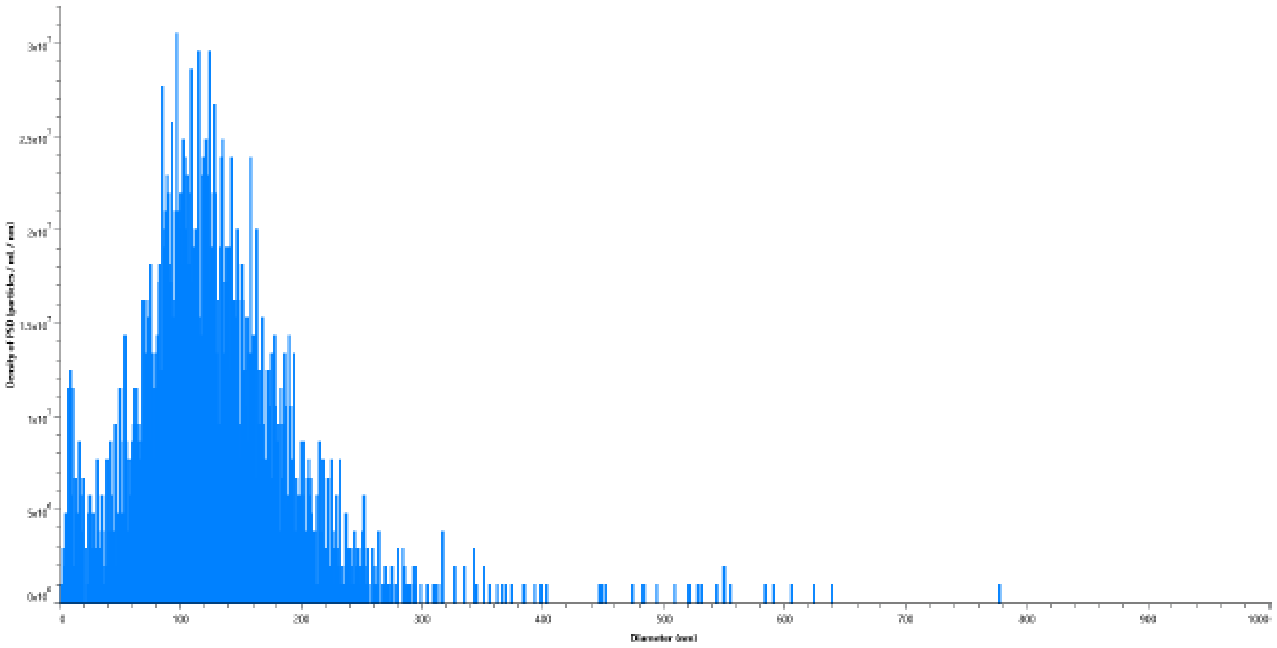

Parameter	Result
**Mean**	136.18 nm
**Mode**	97.50 nm
**D10**	75.41 nm
**D50**	125.89 nm
**D90**	206.03 nm
**Span**	1.038 nm
**Concentration (sample)**	2.49×10^{9} particles/mL
**Concentration (minus blank)**	2.49×10^{9} particles/mL
**Integration range**	50 - 500 nm
**Chart type**	ConstantBinsTable
**Volume calibration**	None

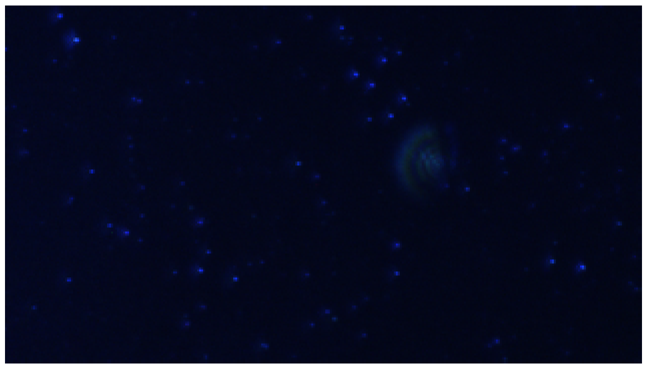

Video processing information

**Detection threshold**	2	**Drift correction**	0%	**Std. dev./CoV**	57 nm / 41.5%
**Detection type**	Polydisperse	**Calibration constant**	197.01 ± 0.1 (nm/pixel)	**Camera type**	AVT Manta 319C
**Cluster algorithm**	Overridden	**Processing date**	2026-03-19 14:28:22	**Volume**	2.10×10^{-6} (mL)
**Feature radius**	40 pixels	**Total/proc. frames**	7500 / 7500	**Software/firmware**	2.61 / 1.3.5
**Tracking starts on**	1 frame	**Total/in range counts**	2875 / 2618	**Serial number**	0052
